# Seasonal meropenem resistance in *Acinetobacter baumannii* and influence of temperature-driven adaptation

**DOI:** 10.1186/s12866-024-03271-y

**Published:** 2024-04-27

**Authors:** Xiaoxuan Liu, Pu Qin, Hainan Wen, Weigang Wang, Jianhong Zhao

**Affiliations:** 1 Hebei Provincial Center for Clinical Laboratories, The Second Hospital of Hebei Medical University, Shijiazhuang, 050000 People’s Republic of China; 2 Department of Laboratory Medicine, Affiliated Hospital of Chengde Medical University, Chengde, 067000 People’s Republic of China

**Keywords:** *Acinetobacter baumannii*, Antibiotic resistance, Seasonality, Meropenem, Temperature

## Abstract

**Background:**

Recognition of seasonal trends in bacterial infection and drug resistance rates may enhance diagnosis, direct therapeutic strategies, and inform preventive measures. Limited data exist on the seasonal variability of *Acinetobacter baumannii*. We investigated the seasonality of *A. baumannii*, the correlation between temperature and meropenem resistance, and the impact of temperature on this bacterium.

**Results:**

Meropenem resistance rates increased with lower temperatures, peaking in winter/colder months. Nonresistant strain detection exhibited temperature-dependent seasonality, rising in summer/warmer months and declining in winter/colder months. In contrast, resistant strains showed no seasonality. Variations in meropenem-resistant and nonresistant bacterial resilience to temperature changes were observed. Nonresistant strains displayed growth advantages at temperatures ≥ 25 °C, whereas meropenem-resistant *A. baumannii* with β-lactamase OXA-23 exhibited greater resistance to low-temperature (4 °C) stress. Furthermore, at 4 °C, *A. baumannii* upregulated carbapenem resistance-related genes (*adeJ*, *oxa-51*, and *oxa-23*) and increased meropenem stress tolerance.

**Conclusions:**

Meropenem resistance rates in *A. baumannii* display seasonality and are negatively correlated with local temperature, with rates peaking in winter, possibly linked to the differential adaptation of resistant and nonresistant isolates to temperature fluctuations. Furthermore, due to significant resistance rate variations between quarters, compiling monthly or quarterly reports might enhance comprehension of antibiotic resistance trends. Consequently, this could assist in formulating strategies to control and prevent resistance within healthcare facilities.

**Supplementary Information:**

The online version contains supplementary material available at 10.1186/s12866-024-03271-y.

## Introduction

*Acinetobacter baumannii*, a nonfermenting gram-negative bacillus, demonstrates robust environmental resilience, enabling survival in dry conditions for several months and susceptibility to clonal transmission [1, 2]. A major contributor to hospital-acquired infections, *A. baumannii* can cause multisite infections in the lungs, bloodstream, and central nervous system [3]. The excessive and irrational use of antibiotics in recent years has led to an increased rate of resistance in *A. baumannii*. Data from the China Antimicrobial Surveillance Network indicate that the resistance rates to first-line drug carbapenems escalated from 13.3 to 73.9% between 2004 and 2018 [4]. *A. baumannii* has strong acquired resistance and is capable of accumulating multiple resistance genes. Once resistance to carbapenems has developed, *A. baumannii* may also develop resistance to other antimicrobial drugs, with the potential emergence of multidrug-resistant (MDR) or even pandrug-resistant strains (PDR) [5–7]. These infections lead to prolonged hospitalization and mortality rates as high as 56-70% [8–12]. In 2018, the World Health Organization (WHO) listed carbapenem-resistant *A. baumannii* (CRAB) as a critical-priority bacterium for the research and development of new antibiotics [6].

The prevalence of infections caused by gram-negative bacteria, such as *A. baumannii*, increases in warm seasons and decreases in cold seasons [13, 14]. This cyclical fluctuation in disease incidence based on seasons or specific time cycles is known as seasonality [15]. Research suggests that bacterial resistance rates also exhibit seasonality [16–18]. For instance, the resistance rates of *Streptococcus pneumoniae* to penicillins and cephalosporins [16] and those of *Klebsiella pneumoniae* and *Escherichia coli* to nitrofurantoin all peak in winter [17, 18].

An accurate understanding of these seasonal trends enables more effective resistance surveillance and serves as a foundation for precise clinical dosing. However, studies on the seasonality of antimicrobial resistance rates of *A. baumannii* are relatively limited^[19]^. Therefore, we retrospectively analyzed the collection month and meropenem resistance rates of clinical isolates from 45 hospitals in Hebei Province between 2020 and 2022. To analyze the seasonality of meropenem resistance in *A. baumannii*, the correlation between meropenem resistance rates and the effect of temperature on *A. baumannii* was determined.

## Materials and methods

### Strain origin, identification, and culture methods

*A. baumannii* resistance data were gathered from 45 hospitals in Hebei Province between January 2020 and December 2022. Duplicate strains isolated from the same patient and site were excluded from the analysis. Bacteria were cultured in Mueller–Hinton (MH) medium or Luria–Bertani (LB) medium (Oxoid; Thermo Fisher Scientific, Waltham, MA, USA) at 37 °C, unless otherwise specified.

### Bacterial identification and antibiotic susceptibility assays

Microbial identification was conducted using automated systems such as VITEK 2 (bioMérieux, Marcy-l’Étoile, France), Phoenix 100 (BD, Franklin Lakes, NJ, USA), and MALDI-TOF (Bruker, Billerica, MA, USA). Antibiotic sensitivity testing was performed using MH medium, and VITEK 2 was also used for this purpose in some hospitals. The antibiotic sensitivity results for quality control strains *E. coli* ATCC 25,922 and *Pseudomonas aeruginosa* ATCC 27,853 consistently fell within the quality control pass range throughout the experimental period. Interpretation of results followed CLSI M100 2022 [19].

### Temperature data sources and seasonal variations

Monthly mean temperatures in Hebei Province, China, from 2020 to 2022 were obtained from ERA5-ground meteorological observations published by the European Center for Medium-Range Weather Forecasts [20].

Isolates were categorized into quarters based on collection dates: Q1 for January through March, Q2 for April through June, Q3 for July through September, and Q4 for October through December. Q1–Q4 corresponded to spring, summer, autumn, and winter, respectively. We also defined May to September (average temperature ≥ 15 °C) as warm months and October to April (average temperature < 15 °C) as cold months.

### Growth assays

Growth assays were performed as described previously [21], with minor modifications. *A. baumannii* from overnight cultures was normalized to an OD_600_ of 0.01 in fresh LB liquid medium and grown at 20 °C, 25 °C, 30 °C, and 37 °C for 24 h with continuous shaking. The OD_600_ was measured every 2 h using a microplate reader (Thermo Fisher Scientific) for 24 h. Maximum specific growth rates (µ_max_) were determined by fitting growth data to a logistic growth curve using GraphPad Prism and the following equation: Y = Y_M_ × Y_0_/ ((Y_M_ − Y_0_) ×e^− xk^ + Y_0_), where Y_0_ and Y_M_ represent OD_600_ values at time points 0 and M, respectively, k is a constant calculated automatically by the program and x represents the time of growth (in hours). Each µ_max_ was calculated as the derivative of the equation for the time point of maximum growth.

### Survival analysis

*A. baumannii* cultured to the logarithmic stage (OD600 ≈ 0.7) at 37 ℃ was transferred to a 4℃ environment. After 0, 12, 24, and 48 h, bacterial solutions were diluted and plated on LB agar. Colony counting determined the survival rate using the formula: survival rate (%) = T_N_/T_0_ × 100%, where T_N_ represents the bacterial count after N hours and T_0_ is the initial count.

### Bacterial conjugation assays

Clinical strain AB7276 (ST1336; *oxa*23^+^; meropenem, MIC > 32 µg/mL; gentamicin, MIC = 1 µg/mL) was chosen as the donor and AB7434 (ST2125; meropenem, MIC = 2 µg/mL; gentamicin, MIC > 8 µg/mL) as the recipient. Logarithmic-phase donor and recipient bacteria cultures (100 µL) were each inoculated into 2 mL of fresh LB medium and incubated at 37 °C for 12 h. The mixed bacterial solution was then inoculated on LB agar medium containing meropenem (4 µg/mL) and gentamicin (8 µg/mL) for transconjugant screening. Colonies that grew in the agar medium containing two antibiotics were preliminarily identified as transconjugants after overnight incubation at 37 °C. A randomly selected colony underwent PCR to confirm it as a positive transconjugant AB7434-poxa23 if it carried *oxa23* and the MLST typing matched that of the recipient.

### Competition experiments in vitro

Competition experiments were conducted in vitro using overnight cultures of strains AB7434-poxa23 and AB7434. Cultures were diluted 1:100 in LB broth and mixed at a 1:1 ratio. Incubation took place at 4 °C, 25 °C, and 37 °C with shaking for 24 h. The mixed populations were diluted and spread on LB agar. The colonies of resistant strains (R) were counted using meropenem (4 µg/mL) plates, and those of nonresistant strains (NR) were counted using plates without antibiotics and subtracted from those obtained with meropenem. The competitive index for the strain AB7434-poxa23 was calculated by dividing the output ratio (R/NR) by the input ratio (R/NR). If the competitive index > 1, the relative fitness of AB7434-poxa23 was considered higher than that of AB7434. The experiment was performed in triplicate.

### Meropenem killing experiments

Meropenem killing experiments were performed as described previously [22], with minor modifications. Log-phase *A. baumannii* was pretreated at 4 °C for 16 h. Cold-treated and untreated log-phase *A. baumannii* were added 1:10 to fresh LB broth containing 10 mg/L meropenem and incubated at 4 °C and 37 °C, respectively. The number of viable organisms was counted at 0, 6, and 24 h. The number of viable organisms after the meropenem killing experiment was determined in the same way as for the survival analysis.

### RNA extraction and quantitative reverse transcription polymerase chain reaction (qRT‒PCR)

*A. baumannii* growing to logarithmic phase at 37 °C was divided into two portions: one was centrifuged at 1000 × g for 3 min to gather the bacterial precipitate, and the other was subjected to a 4 °C cold treatment for 16 h before collecting the bacterial precipitate. The pellets were stored at − 80 °C. RNA extraction was carried out using a total RNA extraction kit (Cat no DP430, TIANGEN, Beijing, China) and was reverse transcribed using a cDNA reverse transcription kit (Cat no R323, Vazyme, Nanjing, Jiangsu, China). The qPCR was performed with initial denaturation at 95 °C for 30 s, followed by 40 cycles of 95 °C for 10 s and 60 °C for 30 s. The 16 S rRNA gene served as the internal reference gene. The primers used for qPCR are listed in S1. Fold-change in gene expression was calculated using the 2^−ΔΔCt^ method [23].

### Statistical analysis

An independent two-sample t test was used to compare differences between groups. Pearson correlation was used to analyze meropenem-resistant and nonresistant bacteria numbers, as well as resistance rate correlation with temperature. Analyses were conducted using WHONET 5.6 (WHO, Geneva, Switzerland) and GraphPad Prism 9.0 (Insightful Science Co. Ltd, San Diego, CA, USA). A *P*-value of ≤ 0.05 denoted statistical significance.

## Results

### Seasonality of meropenem resistance rates

Between January 2020 and December 2022, a total of 27,754 *A. baumannii* were obtained from 45 hospitals in Hebei Province. The mean *A. baumannii* counts were 777 during warm months (May to September) and 676 during cold months (October to April) (95% confidence interval [95% CI]): −181.1 to − 19.84) (*P* = 0.0156). The detected number of *A. baumannii* indicated a seasonal change withincreases in summer and decreases in winter. We assessed the resistance of *(A) baumannii* to various antibiotics, carbapenems, cephalosporins, beta-lactam inhibitors, fluoroquinolones, aminoglycosides, and polymyxin (B) The results showed a relatively slight change in the resistance rates of *A. baumannii* to amikacin and polymyxin, both staying below 16% and 4%, respectively. In addition to this, resistance rates for all antimicrobial drugs that are used commonly in *A. baumannii* showed a cyclic trend, peaking in the first quarter (winter) and reaching a trough in the third quarter (summer) (see S2 in the supplemental material). Carbapenems, crucial for treating MDR bacterial infections, particularly meropenem and imipenem, were significant. CRAB is classified as a priority threat by the CDC due to MDR. Therefore, we focused on meropenem resistance to initially explore seasonal patterns in *A. baumannii* resistance rates.

**Fig. 1:**
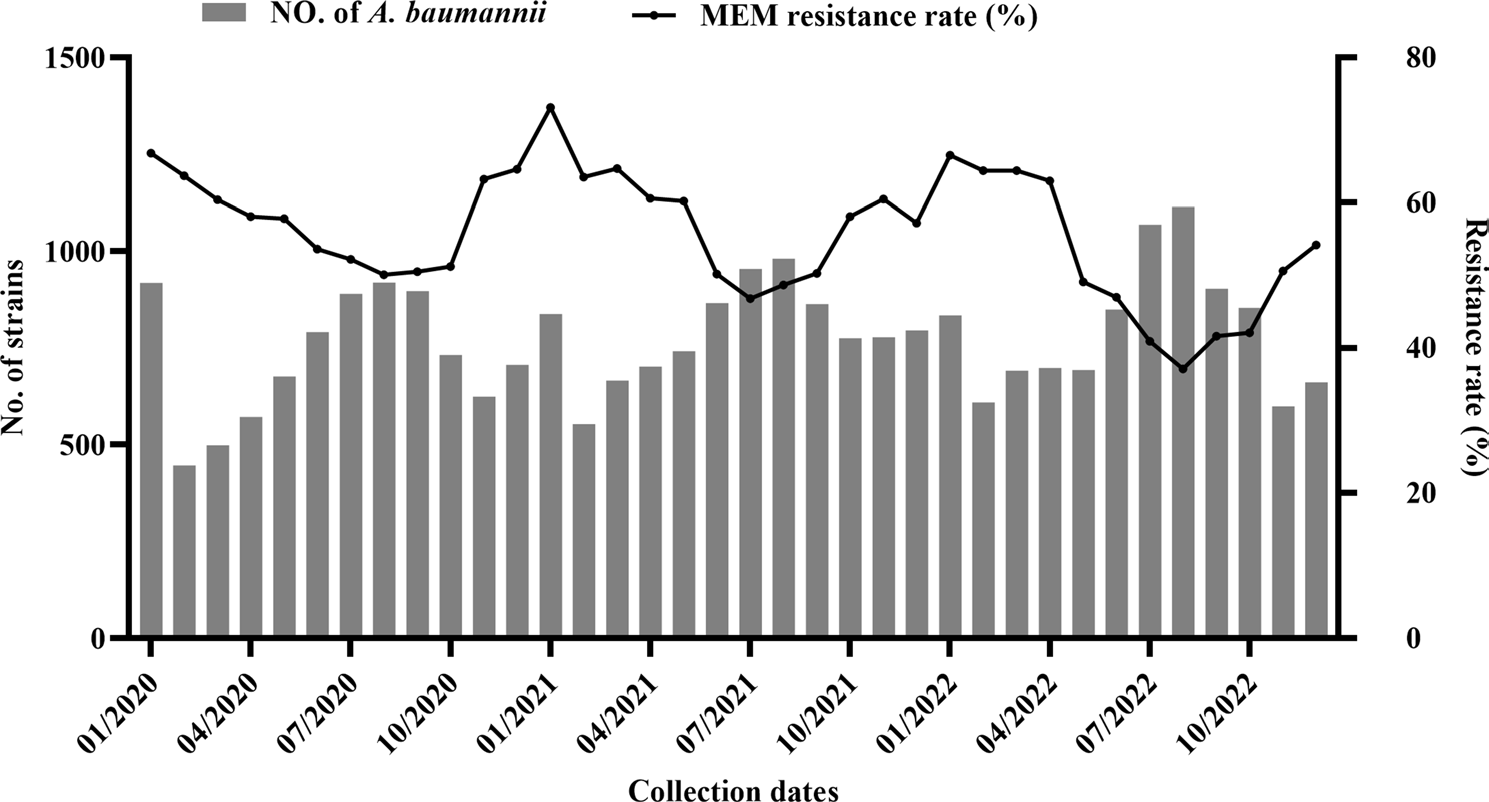
Monthly detection number and meropenem resistance rate of *A. baumannii* in Hebei Province, 2020–2022. The monthly cumulative total (gray bars, left axis) and meropenem (MEM) resistance rates (line chart, right axis) of isolates obtained over the study period

Out of the 27,754 *A. baumannii* strains, 15,201 (54.8%) were meropenem-resistant (MEM^R^), whereas 12,553 (45.2%) were nonresistant (MEM^NR^). Meropenem resistance rates were 64.3%, 67.8%, and 65.2% in the Q1 of 2020 − 2022, respectively; 56.2%, 56.6%, and 52.6% in the Q2; 50.9%, 48.5%, and 37.2% in the Q3; and 59.4%, 58.6%, and 48.3% in the Q4. Resistance rates varied significantly across quarters within the same year, ranging from 13.4 to 28.0%, indicating a clear pattern of seasonality (*P* < 0.0001). We investigated the correlation between resistance rate and local air temperature. The results revealed a negative correlation (*r* = − 0.6862, *P* < 0.0001), indicating that as the temperature decreased, the resistance rate tended to increase (Fig. 1).

### Seasonality of MEM^R^ and MEM^NR^*A. baumannii*

**Fig. 2:**
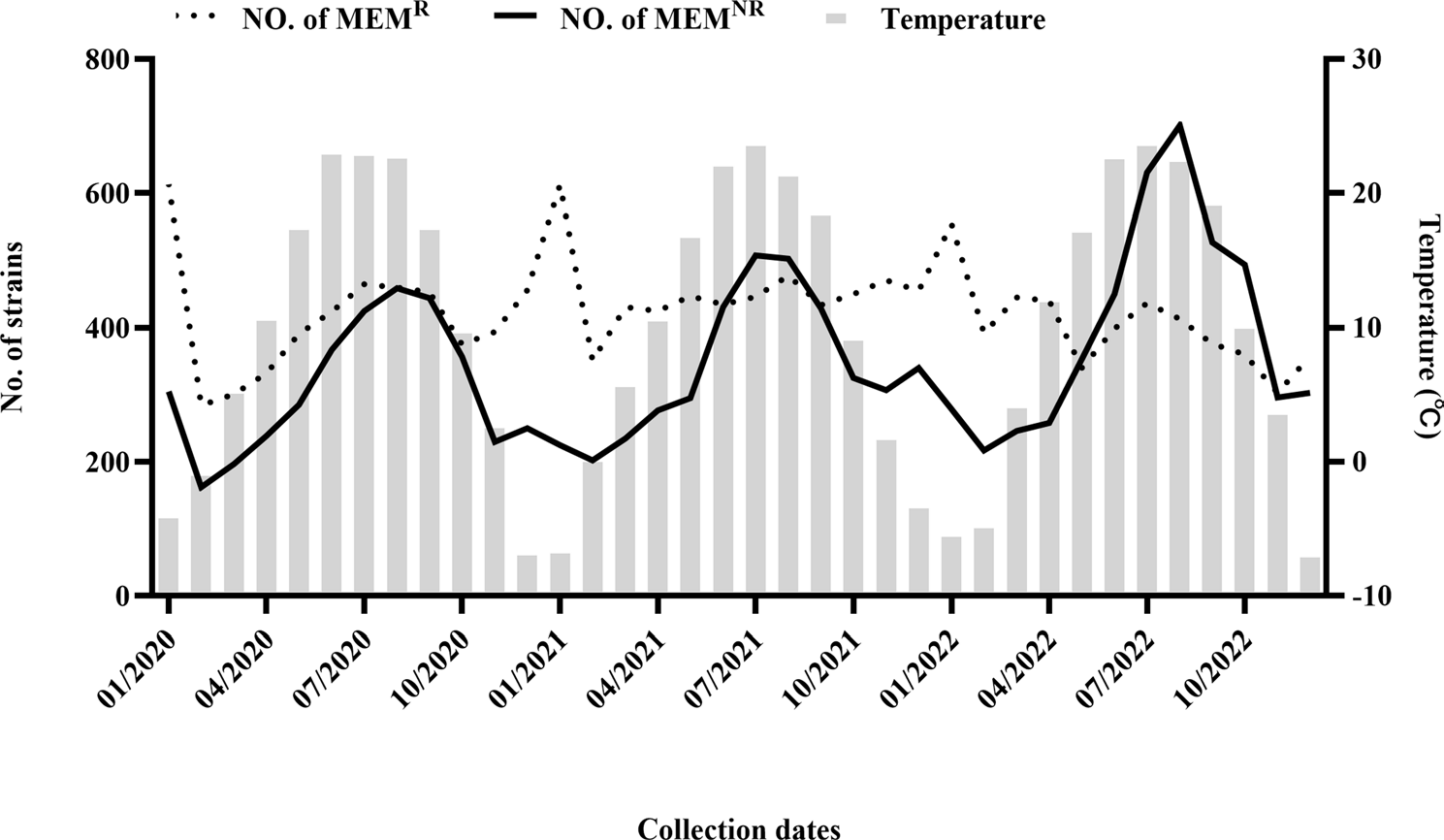
Monthly detection number of MEM^R^ and MEM^NR^*A. baumannii* and local monthly mean temperature in Hebei Province, 2020–2022. The monthly number of MEM^R^ (dotted line) and MEM^NR^ (solid line) *A. baumannii* over the study period (left axis), with the monthly average temperatures in Hebei Province superimposed (gray bars, right axis)

Next, we explored the seasonality and temperature correlation of MEM^R^ and MEM^NR^*A. baumannii* by stratifying the analysis based on meropenem resistance. Figure 2 presents MEM^R^ and MEM^NR^*A. baumannii* counts per month over the last 3 years, juxtaposed with local average monthly temperatures. During warm months, the mean MEM^NR^*A. baumannii* count was 464, contrasting with 274 during cold months (95% CI: −254 to − 127, *P* < 0.001). Strain count was positively linked to temperature (*r* = 0.7215, *P* < 0.0001), exhibiting seasonal increases in summer and decreases in winter. For MEM^R^, the average strain count was 426 in warm months and 419 in cold months (95% CI: −58 to 44, *P* = 0.7740). Strain count remained relatively stable across all quarters and was not correlated with temperature (*r* = 0.0148, *P* = 0.9317), displaying no evident trend in fluctuating. This led us to hypothesize that the seasonality in *A. baumannii* detection number and resistance rate was unrelated to drug-resistant strains but rather stemmed from increased sensitive strains during summer/warm months and decreased strains during winter/cold months.

### Differential adaptation of MEM^R^ and MEM^NR^*A. baumannii* to temperature

**Fig. 3:**
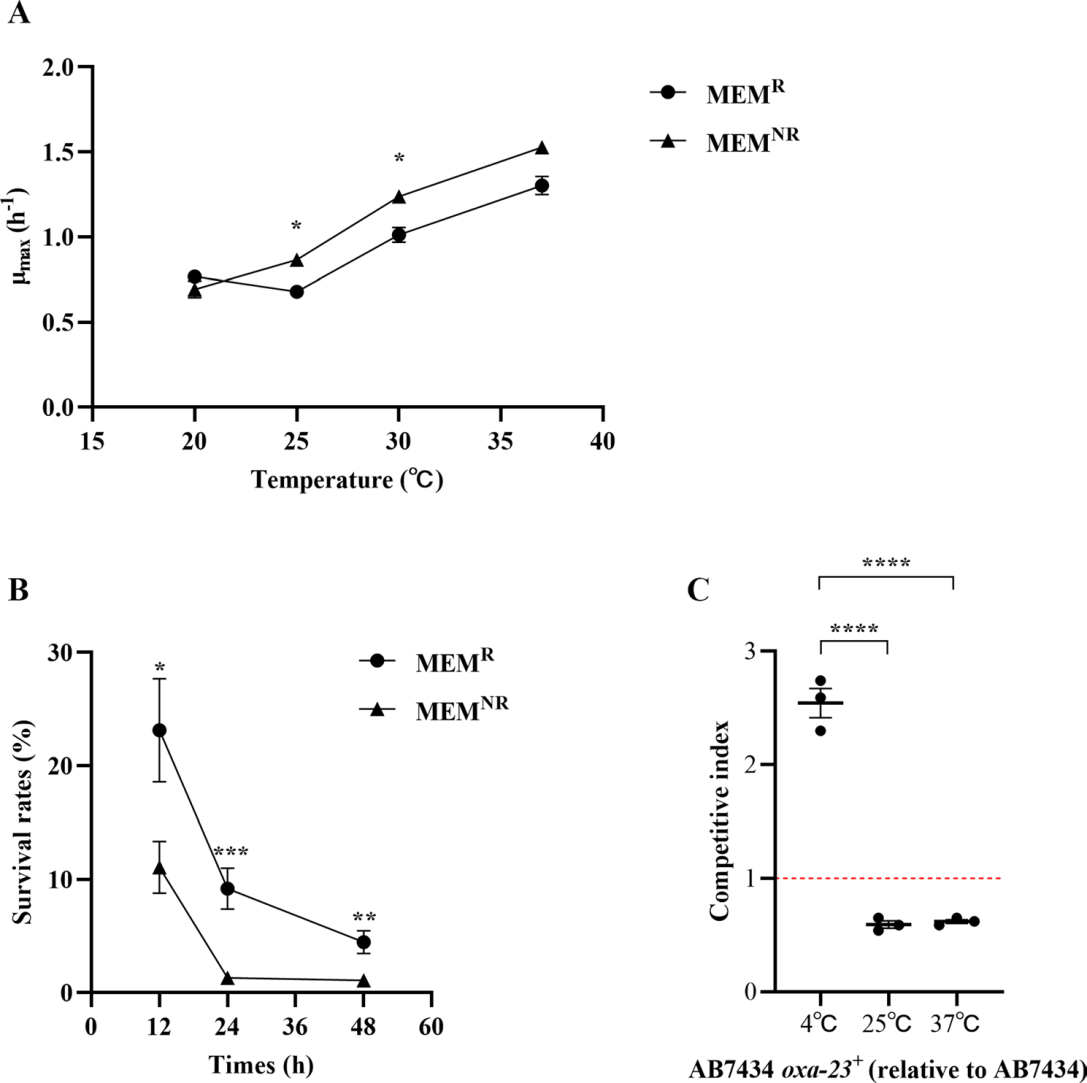
Effect of temperature on the growth kinetics and survival rate of *A. baumannii*. (**A**) Growth kinetics of *A. baumannii* were compared between MEM^R^ (*n* = 13) and MEM^NR^ (*n* = 7) using an independent two-sample t test. (**B**) Survival rates of *A. baumannii* at 4℃ were compared between MEM^R^ (*n* = 2) and MEM^NR^ (*n* = 2) using an independent two-sample t test. (**C**) Competitive index of AB7434*-*poxa23 at 37, 25, and 4 ℃ were compared using one-way ANOVA. Data are presented as the mean ± SEM of *n* = 3 independent experiments. Asterisks denote statistical significance: *P < 0.05, ** *P* < 0.01, *** *P* < 0.001, **** *P* < 0.0001

Temperature changes affect bacterial proliferative capacity and various physiological and biochemical activities [21, 24]. To investigate why MEM^R^ and MEM^NR^*A. baumannii* exhibited varying trends in response to temperature changes. We studied the impact of different temperatures on *A. baumannii* growth. The results in Fig. 3A show that the maximum growth rate (µ_max_) of *A. baumannii* increased with increasing culture temperature (*P* < 0.05). At 20 °C, the MEM^R^ and MEM^NR^ strains demonstrated similar growth rates; as the temperature rose, MEM^NR^ showed a growth advantage with a higher µ_max_. At low temperature, bacterial growth and metabolism were suppressed, and partial bacterial lysis and death were observed [25]. At 4 °C, although the growth of *A. baumannii* was inhibited, MEM^R^ strains had a higher survival rate (Fig. 3B). OXA enzymes, particularly acquired OXA-23, a commonly acquired β-lactamase in *A. baumannii*, lead to bacterial resistance against nearly all β-lactams [7]. The resistant plasmid poxa23, carrying *oxa-23*, was transferred from the resistant strain AB7276 to the sensitive strain AB7434 through conjugation. An in vitro competition assay was then conducted using AB7434-poxa23 and AB7434 to assess the relative fitness of AB7434-poxa23 at 4 °C, 25 °C, and 37 °C through the competitive index. The strain became MEM^R^ (MIC increased from 2 µg/mL to > 32 µg/mL) after plasmid acquisition, but this came at a fitness cost, measured by competitive indexs of 0.59 and 0.62 °C at 25 and 37 °C, respectively. The study found that the fitness cost of *A. baumannii* with poxa23 was reversed in low-temperature environments. Specifically, lowering the incubation temperature to 4 °C resulted in an increased competitive index of 2.54, with AB7434-poxa23 dominating growth (Fig. 3C). The results suggest that the MEM^R^ strains are more adaptable to low-temperature environments than MEM^NR^, possibly due to physiological and metabolic adaptations induced by meropenem resistance.

### Temperature impact on carbapenem-related resistance gene expression in *A. baumannii*

Both meropenem and low-temperature stress impact the membrane structure of *A. baumannii*. Carbapenems, including meropenem, disrupt bacterial cell membranes via inhibition of peptidoglycan (PG) synthases, leading to bacterial lysis and death [26]. Temperature reduction stiffens bacterial cell membranes. Bacteria adapt to temperature changes by adjusting membrane fluidity and permeability [27, 28]. The MEM^R^ strain was more resistant to low-temperature stress, implying that carbapenem-related resistance mechanisms may be involved in low-temperature adaptation in *A. baumannii*.

**Fig. 4:**
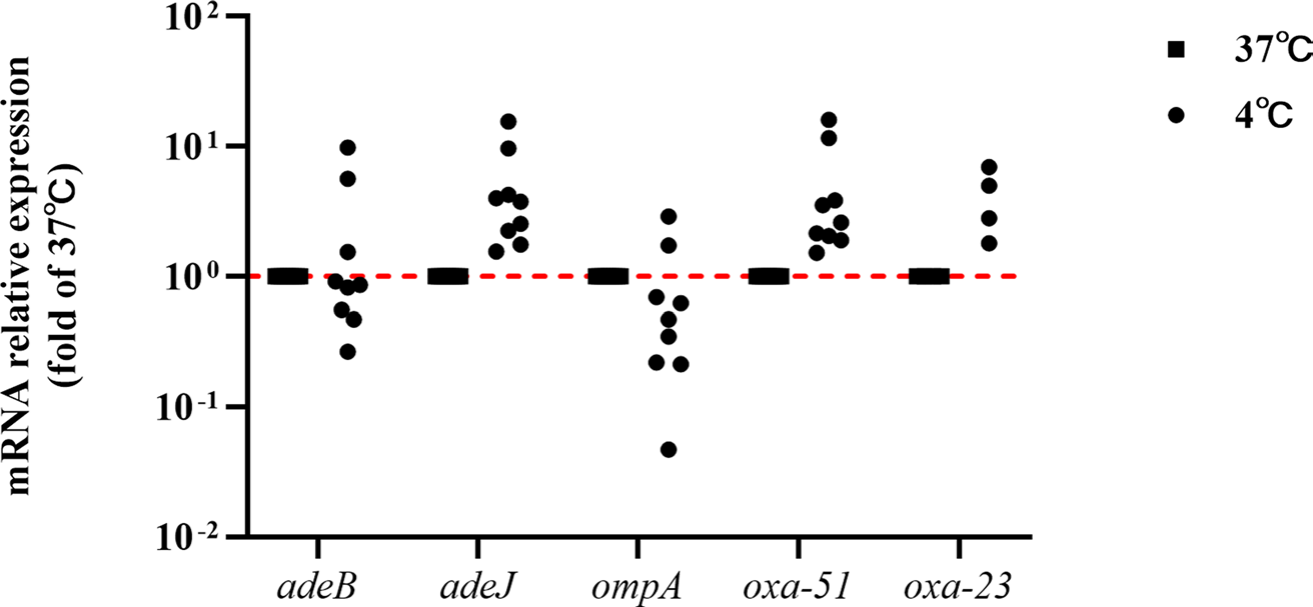
Effect of low-temperature stress on the expression of efflux pump, porin, and β-lactamase encoding genes in *A. baumannii*. Gene expression encoding efflux pumps (*adeJ* and *adeB*), porin (*ompA*), and β-lactamase (*oxa-51* and *oxa-23*) was analyzed in *A. baumannii* ATCC19606 and eight clinical isolates. These isolates included four MEM^R^ isolates carrying *oxa-23* and four MEM^NR^ isolates. Nine strains of *A. baumannii* underwent 16 h at 4 °C. The relative levels of expression of genes (versus 37 °C) for each tested strain are presented as the mean of *n* = 3 independent experiments

Meropenem resistance in *A. baumannii* primarily involves β-lactamases, efflux pump overexpression, and reduced membrane permeability [29, 30]. OXA enzymes, particularly acquired OXA-23, are the principal β-lactamases that dominate carbapenem resistance [7, 29, 31]. OXA-51, inherent in *A. baumannii*, imparts varied carbapenem resistance levels when overexpressed due to promoter insertion [29, 31]. The efflux pumps AdeABC and AdeIJK contribute to multidrug resistance [32–35]. Porin OmpA, a significant virulence factor in *A. baumannii*, interacts with efflux pumps, intensifying the resistance of *A. baumannii* [36, 37]. We selected ATCC19606 and eight clinical strains, consisting of four MEM^R^ strains (carrying oxa-23) and four MEM^NR^ strains, to assess the expression of the common carbapenem resistance-related genes described above (*adeB*, *adeJ*, *ompA*, *oxa-51*, and *oxa-23*) at 4 °C. Figure 4 displays upregulated *adeJ*, *oxa-51*, and *oxa-23* expression across the nine strains after 16 h at 4 °C.The *adeB* and *ompA* genes displayed strain-specific expression, possibly regulated by factors other than temperature. The expression values for each strain and each gene are shown in S3.

### Enhanced meropenem resistance in *A. baumannii* adapted to low-temperature

**Fig. 5:**
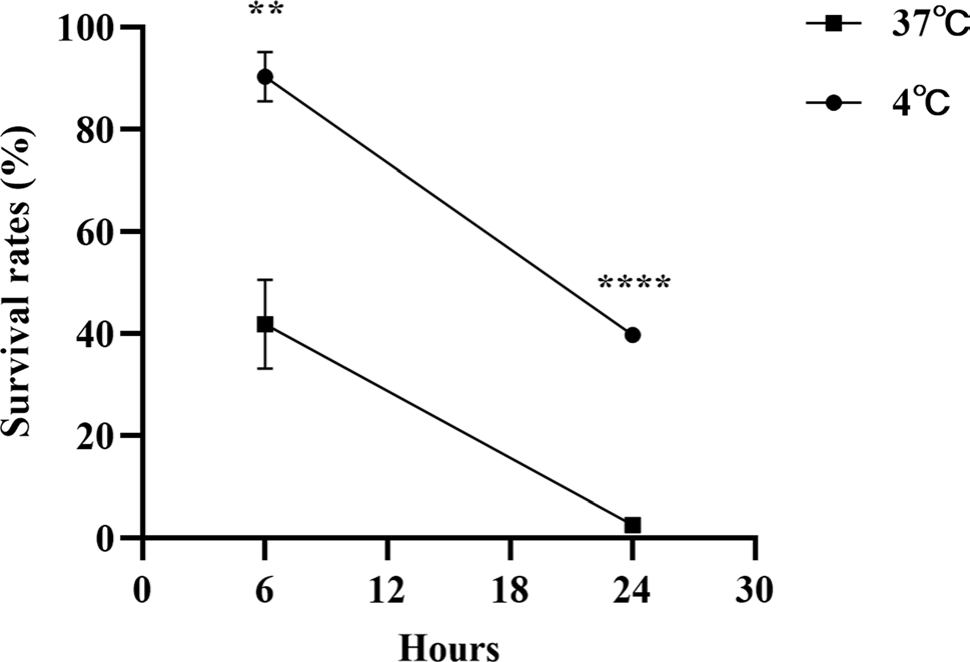
Effect of temperature on the tolerance of *A. baumannii* ATCC19606 to MEM stress. The difference in survival rates of *A. baumannii* ATCC19606 in MEM stress at 37 °C and 4 °C was determined by an independent two-sample t test. All data are presented as the mean ± SEM of *n* = 3 independent experiments. The asterisk distinguishes statistical significance: **P < 0.01, ****P < 0.0001

To verify whether the low-temperature adapted upregulation of meropenem resistance genes can increase the tolerance of *A. baumannii* to meropenem, we assessed the survival of *A. baumannii* ATCC19606 under meropenem stress (10 µg/mL; 20-fold the MIC for *A. baumannii* ATCC19606) at 37 and 4 °C. After 6 h of exposure, the average survival of ATCC19606 was 41.9% at 37 °C and 84.1% at 4 °C. Continued exposure for 24 h reduced survival to 2.6% and 39.8%, respectively (Fig. 5). The tolerance of *A. baumannii* to meropenem stress was notably stronger at 4 °C, as evidenced by its higher survival within 24 h compared to 37 °C. In addition, we also observed that meropenem (0.125 µg/mL; 1/4-fold the MIC for ATCC19606) exposure also upregulated *adeJ* and *oxa-51* gene expression in *A. baumannii* ATCC19606 (Supplemental S4). These results imply a degree of cross-tolerance in *A. baumannii* to both stressors, low temperature and meropenem exposure.

## Discussion

Consistent with previous studies [38], in this study, we observed a seasonal trend in the population of *A. baumannii*, with numbers increasing in summer and decreasing in winter. Kumar et al. [39] observed higher resistance rates in cold regions (− 4 –10 °C) without human intervention than in warm (25–37 °C) and hot (50–60 °C) regions. Interestingly, the meropenem resistance rate of *A. baumannii* also displayed significant seasonal fluctuations, rising with decreasing temperature. The difference in the rate of resistance between quarters of the same year reached 13.4−28.0%. MEM^R^ strains remained stable monthly, therefore, the seasonality of the resistance rate could be due to the increase and decrease of MEM^NR^ strains in summer and winter, respectively [40, 41].

Acquisition of drug resistance often imposes a fitness cost on bacteria, typically evidenced by a slowdown in growth and proliferation [42, 43]. According to this hypothesis, when antimicrobial selective pressure is absent, the viability of resistant strains decreases. Subsequently, sensitive strains gain a growth advantage, leading to the gradual elimination of resistant strains [43]. To simulate human body temperature, the fitness cost is commonly tested at 37 °C. However, environmental factors such as growth conditions and temperature influence the fitness cost, and sensitive strains do not consistently exhibit a growth advantage [44, 45]. For instance, the Inck1 resistance plasmid incurs a higher fitness cost at 42 °C than at 37 °C [45], and a reduced lack of nutrient status decreases the fitness cost caused by the *rpoB* mutation, enabling resistant bacteria to become more competitive [44].In this study, we found that MEM^R^ and MEM^NR^*A. baumannii* had distinct adaptations to varying ambient temperatures. Extensively resistant bacteria seem to show greater adaptability in harsh environments [24]. The survival rate of MEM^R^ was higher than that of MEM^NR^ at 4 °C. MEM^NR^ strains gained a growth advantage at higher temperatures. However, in vitro competition experimental results show that exposure to low temperatures reversed the fitness cost imposed on *A. baumannii* by the drug-resistant plasmid poxa23. At 4 °C, strain AB7434-poxa23 demonstrated a significant competitive advantage.

Both non-optimal temperature and antibiotic exposure act as stressors. Some studies indicate an overlap between cellular processes disrupted by antibiotics and those affected by temperature changes [46, 47]. In this study, a 4 °C low-temperature treatment increased the survival of *A. baumannii* ATCC19606 under meropenem stress. Low temperature and meropenem exposure upregulated the expression of *adeJ* and *oxa-51* [48, 49]. Furthermore, after exposure to a low temperature of 4 °C, we observed an upregulation in the expression of *oxa-23* in four strains of MEM^R^*A. baumannii*. These results suggest that *A. baumannii* exhibits a certain degree of cross-adaptation to the exposure of both low temperature and meropenem, and genes *adeJ*, *oxa-51*, and *oxa-23*, which are associated with meropenem resistance, appear to be involved. Similar findings have been observed in other studies, demonstrating the upregulation of AmpC, OXA-51, and AdeIJK under desiccation stress [1]. AdeIJK and the β-lactamase family protein ABUW_2123 are essential for the *A. baumannii* colonization of *G. mellonella* [50]. Bacteria that have extensive resistance seem to demonstrate greater adaptability in harsh environments [24].Based on these findings, we hypothesized that AdeIJK, OXA-51, and OXA-23 not only confer antibiotic resistance to *A. baumannii* but also aid its survival in harsh environments, such as low temperatures.

The cell membrane of gram-negative bacteria is a dynamic barrier consisting of three parts: the inner plasma membrane, the periplasm, and the outer membrane (OM) [27]. The OM is an asymmetric bilayer consisting mainly of lipopolysaccharides or lipo-oligosaccharides in the layers of the OM and glycerophospholipids in the layers of the inner membrane, which protects the bacterium against environmental stresses, such as host-defense factors and antibiotics [51]. *A. baumannii* regulates the lipid composition and fluidity of membranes, enhancing short-chain fatty acid or unsaturated fatty acid content while reducing rigidity to maintain the normal function of the cytosol in low-temperature environments [27, 28]. The study found that the AdeIJK efflux pump is involved in endogenous fatty acid transport [52, 53]. In *A. baumannii*, overexpression of the AdeIJK pump results in a decrease in glycerophospholipids containing longer-chain fatty acids (> 32 carbons) and an increase in shorter-chain glycerophospholipids [54]. Furthermore, deleting AdeIJK leads to inadequate lipid α-acylation in *A. baumannii* [53]. For gram-negative bacteria such as *A. baumannii*, lipid A acetylation or octanoylation may be used as a cold acclimatization strategy to preserve membrane fluidity and permeability [51, 55]. Therefore, we hypothesized that AdeIJK is involved in cold acclimatization in *A. baumannii* by altering the composition of the cell membrane.

PG resides within the periplasmic space, encircling the outer cytoplasmic membrane in a reticular formation, which is vital for upholding cellular morphology and structure [56]. β-lactamases, originating from penicillin-binding proteins, can also attach to PG substrates and maintain their function, albeit to a lesser extent than their Penicillin binding proteins relatives [7, 42, 57, 58]. For instance, the increased PG amidase activity associated with *A. baumannii* OXA-23 overexpression leads to changes in PG composition and an overall increase in cross-linking [42]. PG exists in two primary cross-linking forms, with the majority being 4 − 3 cross-links and a smaller proportion being 3–3 cross-links [56]. A heightened presence of 3–3 cross-links is crucial for upholding PG integrity and for preventing bacterial rupture during environmental stresses such as low temperatures and antibiotic exposure [56, 59, 60]. Endogenous ADC-7 β-lactamase from *A. baumannii* demonstrates L, D-transpeptidase activity, whereby its overexpression boosts 3–3 cross-linked PG synthesis [57]. L, D-transpeptidase is a key enzyme in the 3–3 cross-linked PG synthesis, and its increased expression has been observed with low temperatures or meropenem exposure [48, 59]. Based on these findings, we propose that β-lactamases OXA-51 and OXA-23 might participate in the PG cycle, upholding cell membrane integrity in *A. baumannii* at low temperatures. It is necessary to further investigate the specific roles of AdeIJK, OXA-51, and OXA-23 in resistance to low-temperature stress and their significance in maintaining cell membrane integrity.

Climate change may impact the spread and evolution of antibiotic resistance [61], yet there are limited studies on the mechanisms of low-temperature adaptation in *A. baumannii*. In the future, we need an extensive exploration of the shared signals or cross-response pathways produced by *A. baumannii* in response to environmental stressors such as desiccation, temperature, and antibiotics, which will provide a reference for controlling the development of antibiotic resistance and the development of new drugs.**Conclusion**.

Meropenem resistance rates in *A. baumannii* display seasonality and are negatively correlated with local temperature, with rates peaking in winter. This is possibly linked to the differential adaptation to temperature fluctuations between resistant and nonresistant isolates. Furthermore, due to significant resistance rate variations between quarters, throughout the year, the use of monthly or quarterly reports might enhance comprehension of antibiotic resistance trends. Consequently, this can assist in formulating strategies to control and prevent resistance within healthcare facilities.

### Electronic supplementary material

Below is the link to the electronic supplementary material.

Supplementary Material 1

## Data Availability

The datasets analyzed during the current study are available from the corresponding author upon request.

## Abbreviations

MH: Mueller-Hinton
LB: Luria–Bertani
µ_max_: maximum specific growth rates
MEM: meropenem
MEM^R^: meropenem-resistant
MEM^NR^: meropenem-nonresistant
MIC: minimum inhibitory concentration
MLST: multilocus sequence typing
CRAB: Carbapene-resistant *Acinetobacter baumannii*
MDR: Multidrug-resistant
XDR: Extensively Drug ResistantOM: outer membrane
PG: peptidoglycan
