# Supplementary material for: Seasonal meropenem resistance in Acinetobacter baumannii and influence of temperature-driven adaptation
Source: BMC Microbiol. 2024 Apr 27;24:149. doi: 10.1186/s12866-024-03271-y (PMC11055336; doi:10.1186/s12866-024-03271-y)
Supplement: Supplementary file 1 — Supplementary Material 1 [file 12866_2024_3271_MOESM1_ESM.docx]

S 1 Table. Primer used in this study

| Primer name | Sequence（5’-3’） |
| --- | --- |
| oxa-23 qF | GCTCTAAGCCGCGCAAATAC |
| oxa-23 qR | GACCTTTTCTCGCCCTTCCA |
| oxa-51 qF | AGCTTCCGCTATTCCGGTTT |
| oxa-51 qR | AAGGACCCACCAGCCAAAAA |
| ompA qF | TCGTGCTATGAACCGTCGTGTATTC |
| ompA qR | CTGCCGCTTCTTGACCAGGTTG |
| adeB qF | ACAAGACCGCGCTAACTTAGGT |
| adeB qR | TGCCATTGCCATAAGTTCATCT |
| adeJ qF | AGCTGGTGCTATGGGCGTTA |
| adeJ qR | GCCACCCCATGCAATACG |
| 16s rRNA qF | ACGCGAAGAACCTTACCTGG |
| 16s rRNA qR | CCCAACATCTCACGACACGA |


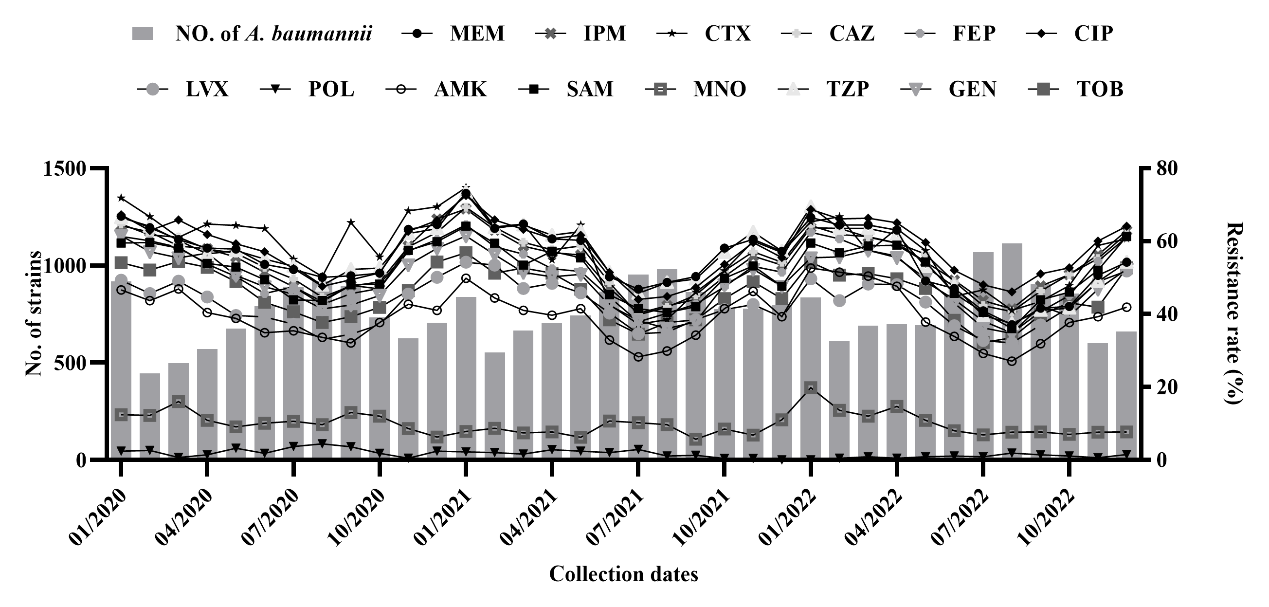


S 2 Figure. Monthly detection number and antibiotic resistance trends of *A. baumannii* in Hebei Province, 2020–2022

The monthly cumulative total (bars, left axis) and antibiotic resistance (line chart, right axis) of isolates obtained over the study period. MEM: Meropenem; IPM: Imipenem; CTX: Cefotaxime; CAZ: Ceftazidime; FEP: Cefepime; CIP: Ciprofloxacin; LVX: Levofloxacin; POL: Polymyxin B; AMK: Amikacin; SAM: Ampicillin/Sulbactam; MNO: Minocycline; TZP: Piperacillin/Tazobactam; GEN: Gentamicin; TOB: Tobramycin.

S 3 Table. The relative expression of efflux pump, porin, and β-lactamase encoding genes in *A. baumannii* at 4℃

| Strains | *adeB* |  | *adeJ* |  | *ompA* |  | *oxa-51* |  | *oxa-23* |
| --- | --- | --- | --- | --- | --- | --- | --- | --- | --- |
| AB6859 | 0.26±0.01 |  | 1.75±0.05 |  | 0.04±0.00 |  | 2.59±0.05 |  | 1.79±0.05 |
| AB6997 | 0.86±0.04 |  | 4.01±0.22 |  | 0.62±0.04 |  | 3.54±0.21 |  | 2.81±0.15 |
| AB7001 | 0.55±0.04 |  | 2.54±0.09 |  | 1.73±0.12 |  | 1.52±0.05 |  | / |
| AB7222 | 0.91±0.07 |  | 9.63±0.75 |  | 0.69±0.02 |  | 16.0±1.09 |  | 6.95±0.97 |
| AB7276 | 1.53±0.12 |  | 2.24±0.08 |  | 2.88±2.58 |  | 1.89±0.19 |  | 4.98±0.17 |
| AB7285 | 9.80±0.59 |  | 15.4±0.31 |  | 0.47±0.15 |  | 11.6±0.51 |  | / |
| AB7644 | 5.66±0.27 |  | 4.25±0.32 |  | 0.21±0.02 |  | 2.05±0.18 |  | / |
| AB7973 | 0.47±0.06 |  | 1.55±0.04 |  | 0.22±0.00 |  | 2.14±0.14 |  | / |
| ATCC19606 | 0.82±0.08 |  | 3.74±0.65 |  | 0.34±0.04 |  | 3.84±0.52 |  | / |

Note: Each isolate was tested three times. The gene relative expression (versus 37°C) for each isolate is presented as mean ± SEM.


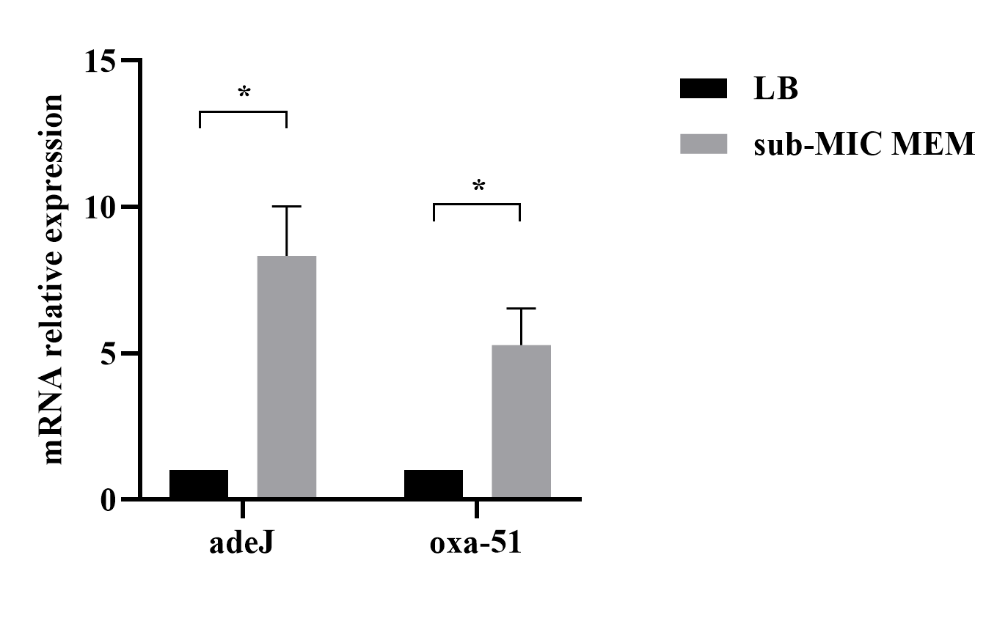


S 4 Figure. Exposure to meropenem promotes the expression of *oxa-51* and *adeJ* in *A. baumannii*

Gene expression was analyzed in *A. baumannii* ATCC19606. The independent two-sample t test was used to compare the relative mRNA expression levels of *A. baumannii* before and after treatment with meropenem. All data are presented as mean ± SEM of n = 3 independent experiments. Asterisks distinguish statistical significance: **P* < 0.0001.
